# Rare Presentation of a Viable Secondary Abdominal Ectopic Pregnancy in the Rudimentary Horn of a Bicornuate Uterus: A Case Report

**DOI:** 10.1002/ccr3.71788

**Published:** 2026-01-04

**Authors:** Shazia Fakhar, Irram Asghar, Atif Rana, Rida Inam, Tehreem Zahid

**Affiliations:** ^1^ Shifa International Hospitals Ltd Islamabad Pakistan; ^2^ Shifa College of Medicine Islamabad Pakistan

**Keywords:** abdominal pregnancy, antepartum hemorrhage, ectopic pregnancy, rudimentary horn

## Abstract

An advanced abdominal in a rudimentary horn pregnancy is rare and difficult to diagnose, especially in late gestation. Accurate early recognition, multidisciplinary planning, and preoperative vascular interventions are critical. With precise surgical management, even in high‐risk scenarios, favorable maternal and neonatal outcomes can be achieved despite significant complications.

## 1 Introduction

Abdominal pregnancies are a rare occurrence and they have been sparsely recorded in literature [1, 2, 3]. Abdominal pregnancy is defined as a pregnancy that has extrauterine implantations in the omentum, critical organs, or major arteries [4]. Abdominal ectopic pregnancies implant within the peritoneal cavity, whereas retroperitoneal pregnancies implant behind the peritoneum, often adjacent to major vessels and retroperitoneal organs [5].

Abdominal pregnancies sometimes occur in the noncommunicating rudimentary horn of the uterus. They are difficult to diagnose and manage as their rupture leads to life‐threatening intraperitoneal hemorrhage. The incidence of rupture of rudimentary horn pregnancy is 1:40,000 [6] and typically occurs between 14 and 20 weeks of gestation. The fetus is then extruded into the abdominal cavity, and the placenta attaches to the pelvic peritoneum and nearby organs, which makes it difficult for the fetus to reach full‐term [7].

Uterine deformity, assisted reproduction, endometriosis, pelvic inflammatory disease, and expectant management of tubal ectopic or salpingostomy are well‐known risk factors for such ectopic pregnancies as they produce tubal anatomic abnormalities and modify the physiological implantation process of the embryo [2]. The location of placental implantation and the availability of vascular supply are thought to be factors that determine the likelihood of embryonic survival.

Diagnosing uterine horn pregnancy is difficult, especially in the early stages, when usual symptoms may be absent or delayed until the second trimester. Once an abdominal pregnancy is diagnosed, an open laparotomy is frequently required to gain greater access to treat placental attachment and reduce bleeding. Uterine horn/cornual pregnancy resembles interstitial tubal pregnancy in several ways, and they can be confused during surgery. The round ligament insertion, which is always lateral to the uterine horn/cornual pregnancy, is a distinctive feature [3]. Abdominal pregnancy to term with a healthy live fetus is thus a very rare condition, with only a few occurrences reported in the past years [4]. Our report highlights this rare occurrence and sheds light on the need for a robust, multidisciplinary team to manage such complex cases.

### 1.1 Informed Consent Statement

Written Informed Consent was obtained from the patient before publication of the case report.

## 2 Case History and Examination

This case report was written after ethical approval (Institutional Review Board: reference number 481‐24) and written informed consent from the patient. The patient was a 23‐year‐old primigravida who conceived spontaneously. She was referred to our tertiary care facility at 28 weeks of gestation from a peripheral hospital, where an anomaly scan had reported an abdominal ectopic pregnancy. Earlier records were unavailable, but according to the patient, the initial antenatal scan had suggested a bicornuate uterus with pregnancy in the right horn. At 24 weeks, she experienced abdominal pain and subsequent imaging (ultrasound and magnetic resonance imaging) confirmed an intraabdominal pregnancy with an empty uterus. She was given steroids for fetal lung maturation and referred for specialized feto‐maternal surveillance and neonatal support.

Her antenatal care commenced at 28 weeks under a multidisciplinary team including neonatology, radiology, and vascular surgery. She had no classical risk factors for abdominal pregnancy. The only comorbidity was gestational diabetes, controlled with metformin. She was counseled extensively about potential complications, including hemorrhage, hysterectomy, organ injury, prolonged intensive care unit (ICU) stay, prematurity, and neonatal morbidity. Despite the risks, the couple opted to continue the pregnancy.

At 30 weeks, she developed persistent flank and right iliac fossa pain. Obstetric ultrasound suggested a bicornuate uterus with thin myometrium and a fetus in transverse lie. Engorged vessels inseparable from uterine walls and lobulated placenta were also noted. MRI at 32 weeks confirmed abdominal pregnancy with empty uterine cavity and markedly engorged vessels, but differentiation from a rudimentary horn pregnancy remained uncertain.

## 3 Differential Diagnosis, Investigations and Treatment

**FIGURE 1:**
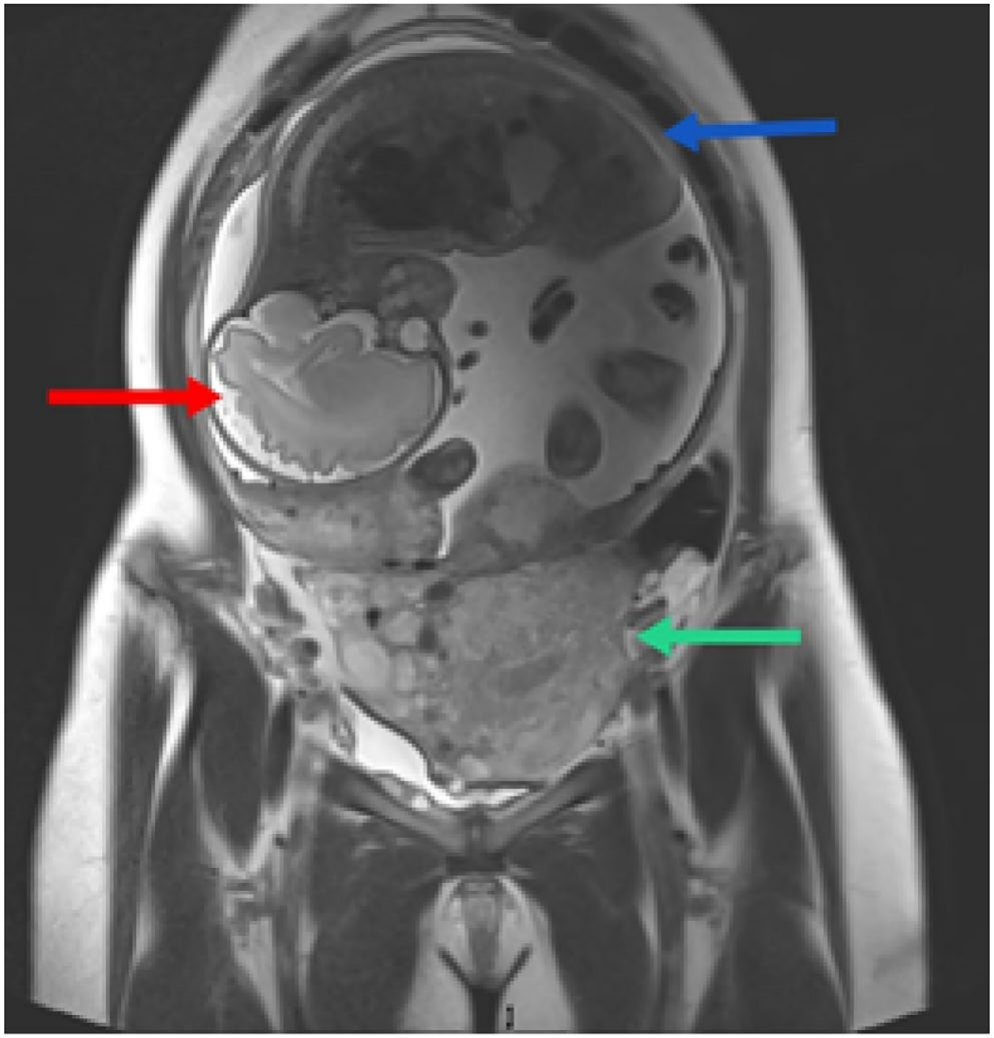
MRI cornual view.

**FIGURE 2:**
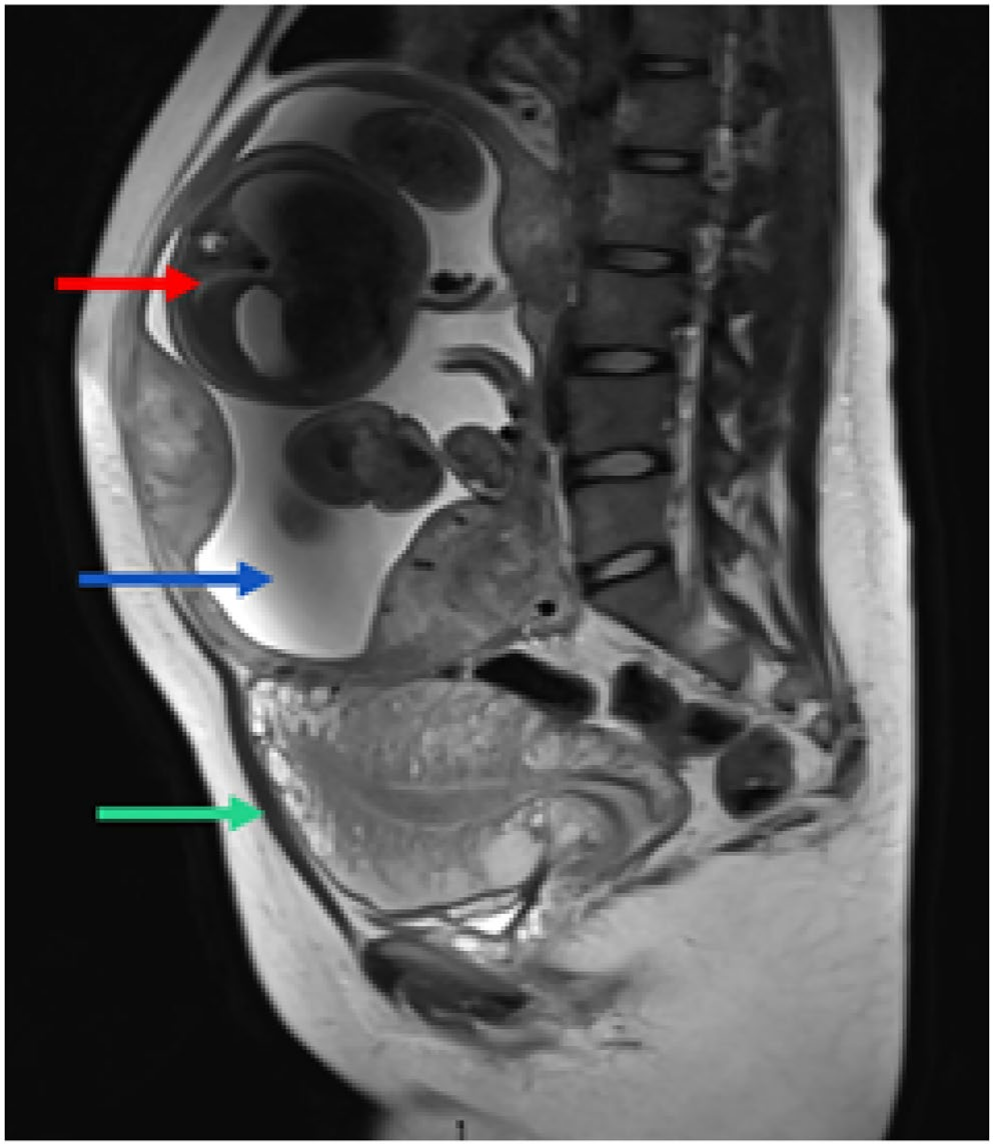
MRI sagittal view. MRI showing an abdominal pregnancy (with gestational age of 32 weeks as per LMP) with empty uterus (green arrow) normally visualized in the pelvis, without any definite communication of the fetal sac with the uterus/cervix with the fetus (red arrow) and placenta (blue arrow) implanted in the abdomen. Sagittal view shows multilobulated placenta located in the anterior and posterior segments.

The differential diagnoses included abdominal pregnancy, cornual pregnancy, and pregnancy in a rudimentary uterine horn. Repeated radiological investigations (ultrasound and MRI) failed to conclusively establish the origin due to advanced gestational age and distorted anatomy (Figures 1 and 2). At 33 weeks, the patient was admitted for planned laparotomy. Preoperative preparations included antenatal steroids, magnesium sulfate for fetal neuroprotection, blood and blood product arrangement, and multidisciplinary surgical planning. Prophylactic bilateral common iliac artery ballooning and DJ stenting were performed to reduce the risk of hemorrhage and urological complications.

**FIGURE 3:**
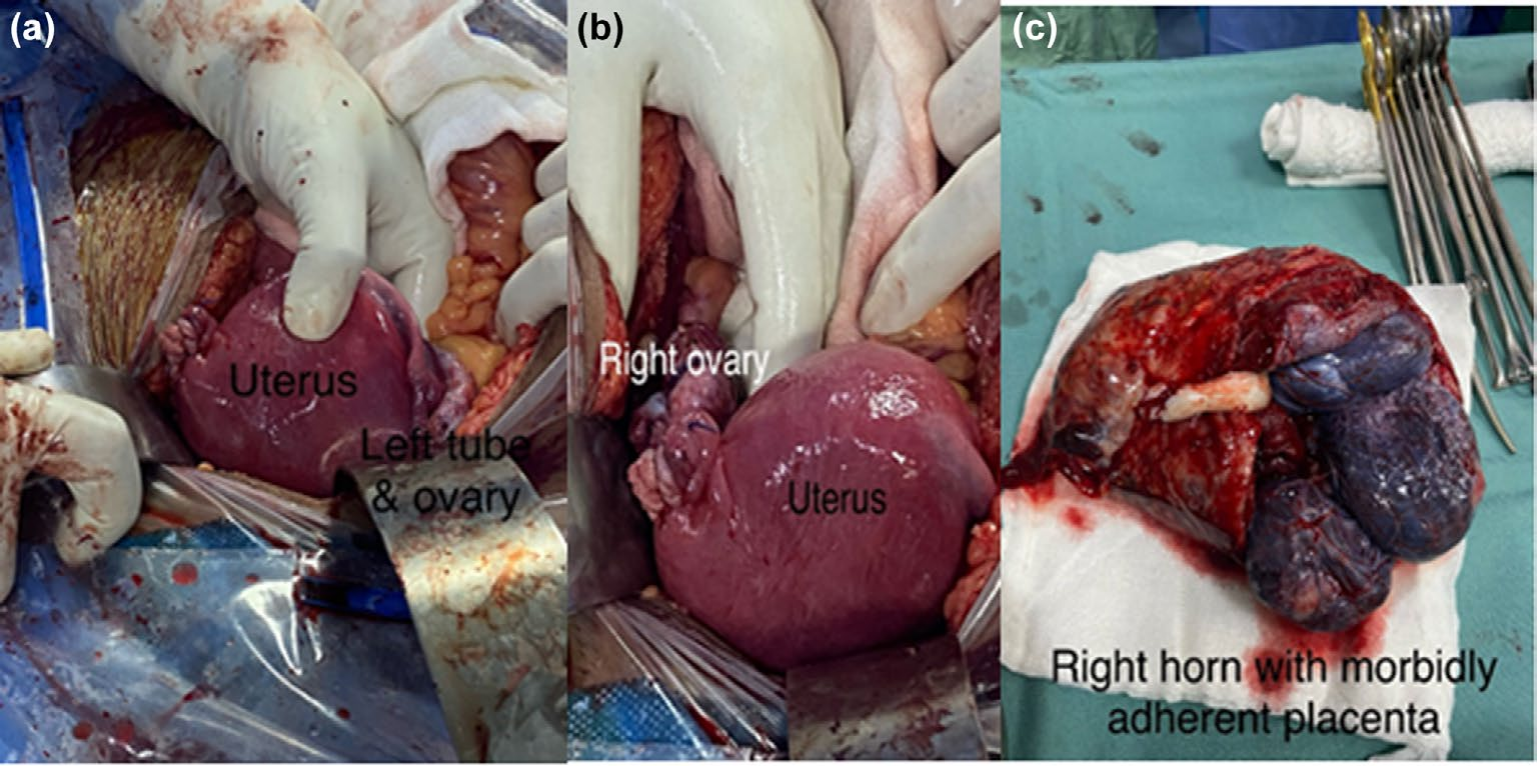
Images before delivery of the baby.

**FIGURE 4:**
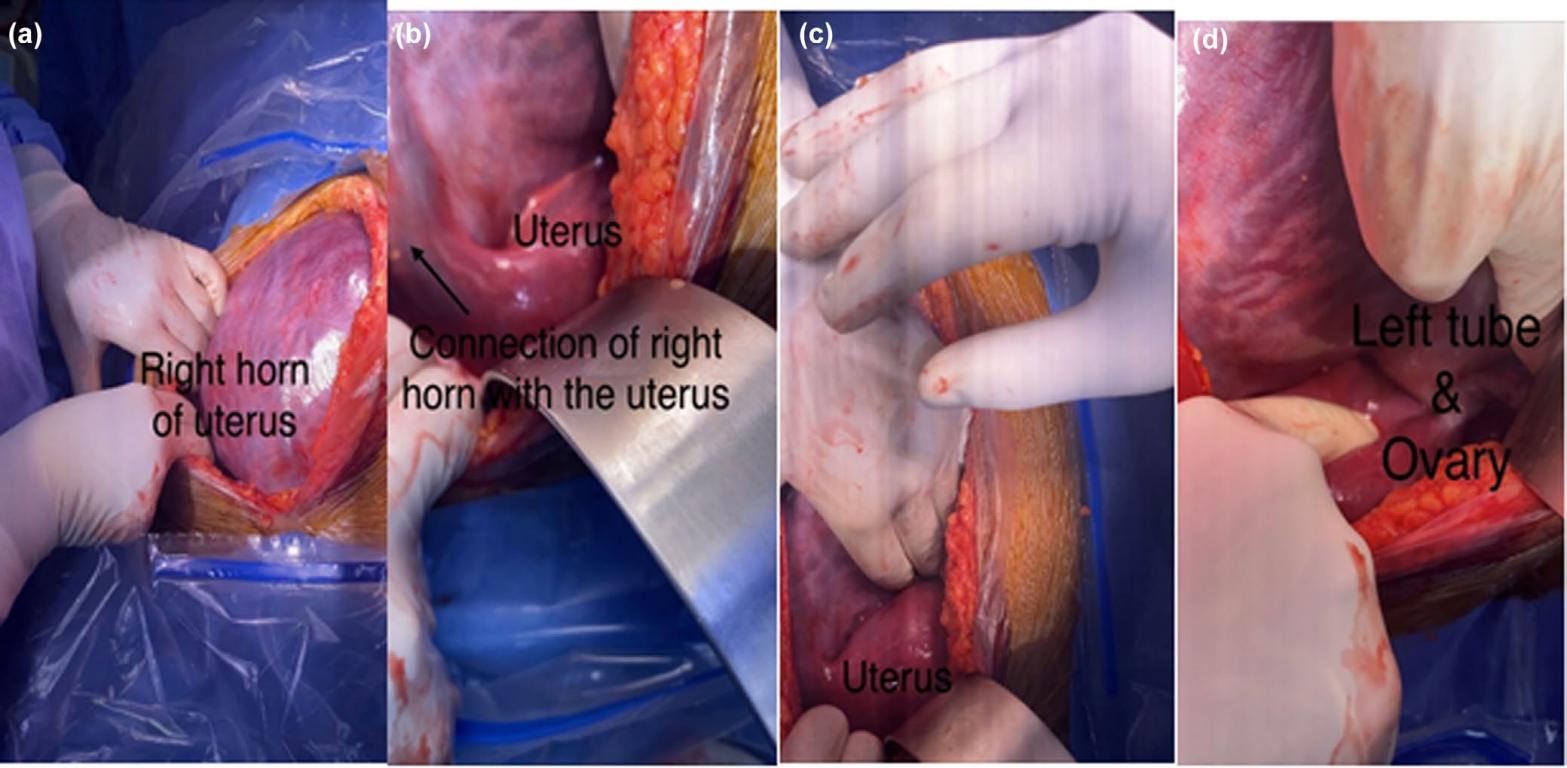
Images after delivery of the baby and removal of rudimentary horn.

Under general anesthesia, a midline laparotomy was performed. The pregnancy sac filled the abdominal cavity with placental tissue extending caudally and communicating with the right horn of the uterus. The uterus was displaced leftward, approximately 12 weeks' size. A vascular clamp was applied at the horn base, and a transverse incision delivered a live baby girl weighing 1.41 kg in breech presentation with Appearance, Pulse, Grimace, Activity, and Respiration score (APGAR) scores of 5 and 7 at 1 and 5 min. The right horn was highly vascular with morbid placental adherence (Figure 3). The rudimentary horn and right fallopian tube were excised, conserving the right ovary. The round ligament was plicated. Estimated blood loss was 1200 mL, necessitating transfusion of 3 packed red cell units. Histopathology confirmed a uterine horn with placenta and right fallopian tube. Chorionic villi showed focal infarction and dystrophic calcification. The resected horn had gestational hyperplasia, and the fallopian tube was unremarkable with no malignancy (Figure 4).

## 4 Conclusion and Results (Outcome and Follow‐Up)

Postoperatively, the patient was monitored in the surgical ICU and recovered uneventfully. Internal iliac balloons and Double J (DJ) stents were removed on postoperative day one. She was discharged in stable condition on day two. The neonate developed moderate respiratory distress which settled with oxygen support, followed by Neonatal Intensive Care Unit (NICU) admission for prematurity and feed rehabilitation. She was discharged after 5 days in stable condition. This case demonstrates the challenge of distinguishing advanced abdominal pregnancy from a rudimentary horn pregnancy. Despite limitations of imaging, careful multidisciplinary planning, preoperative vascular interventions and prompt surgical management ensured favorable maternal and neonatal outcomes.

## 5 Discussion

Most of the cases of abdominal pregnancies are secondary from aborted or ruptured tubal pregnancy [8, 9, 10], however, our case was rare as it started as a rudimentary horn ectopic pregnancy which secondarily became abdominal, remained partially cornual as covered by myometrium at the proximal part and received its blood supply from there. Heterotopic pregnancies with interstitial sites have also been reported with complex multidisciplinary management [10]. A review of similar cases is given in Table 1.

**TABLE 1:** Literature review of similar cases.

Year	Patient demographics	Gestational age at presentation	Clinical presentation	Diagnosis and management	Outcome
2017	Young multiparous female	Not specified (rupture, 2nd trimester typical)	Acute abdomen, shock	Laparotomy, excision of noncommunicating rudimentary horn, ipsilateral salpingectomy	Uneventful recovery, discharged day 8 [11]
2015	28‐year‐old, previous C‐section	20 weeks	Severe abdominal pain, hypotension, internal bleeding	Laparotomy, excision of accessory horn	Recovered after transfusion [12]
2017	23‐year‐old, prior full‐term delivery	~12 weeks (3 months amenorrhea)	Shock, abdominal pain	Laparotomy, excision of right rudimentary horn, right salpingectomy	Discharged after 10 days [13]
2021	26‐year‐old, gravida 4 para 2	16 weeks	Severe pain, shock, anemia, hemoperitoneum	Laparotomy, horn preserved, uterus repaired	Uneventful post‐op
2021	Primigravida	20 weeks (twin pregnancy)	Ruptured ectopic, emergency	Laparotomy, excision of right horn	Advised to avoid pregnancy for 1 year [14]

There are reports of fetal malformations as high as 40% associated with abdominal pregnancies and only 50% of these babies survive up to 1 week post delivery [15, 16] however, in our case, a baby girl weighing 1.41 kg was delivered as breech with no fetal anomalies seen on serial ultrasounds. It is extremely uncommon for such cases to result in a viable baby. These cases usually result in the rupture of the horn in the second or third trimester, typically between the 10th and 20th week of gestation, although a rupture has been reported at 34 weeks [17, 18]. In the majority (83%) of cases, the rudimentary horn is noncommunicating [11] however, in this case, a clear communication between the right horn containing the pregnancy and the uterus was identified. Close liaison with a multidisciplinary team, continuous feto‐maternal surveillance, and good antenatal and postnatal care were the cornerstones in successfully managing this case.

## Author Contributions

**Shazia Fakhar:** conceptualization, funding acquisition, investigation, resources, supervision, visualization. **Irram Asghar:** data curation, formal analysis, methodology, validation, writing – original draft. **Atif Rana:** investigation, supervision, validation. **Rida Inam:** data curation, methodology, visualization, writing – original draft. **Tehreem Zahid:** project administration, writing – review and editing.

## Funding

The authors have nothing to report.

## Conflicts of Interest

The authors declare no conflicts of interest.

## Data Availability

As it is a case report, no datasets were required for this manuscript. Additional information about the case can be furnished on request.
